# Recent demographic histories of temperate deciduous trees inferred from microsatellite markers

**DOI:** 10.1186/s12862-021-01805-w

**Published:** 2021-05-18

**Authors:** Yu Cao, Da-Yong Zhang, Yan-Fei Zeng, Wei-Ning Bai

**Affiliations:** 1 State Key Laboratory of Earth Surface Process and Resource Ecology and Ministry of Education Key Laboratory for Biodiversity Science and Ecological Engineering, College of Life Sciences, Beijing Normal University, Beijing, 100875 China; 2 State Key Laboratory of Tree Genetics and Breeding, Chinese Academy of Forestry, Beijing, 100091 China

**Keywords:** Asian butternuts, Demographic inference, Effective population size, MIGRAINE, Oaks, VarEff

## Abstract

**Background:**

Accurate inference of demographic histories for temperate tree species can aid our understanding of current climate change as a driver of evolution. Microsatellites are more suitable for inferring recent historical events due to their high mutation rates. However, most programs analyzing microsatellite data assume a strict stepwise mutation model (SMM), which could cause false detection of population shrinkage when microsatellite mutation does not follow SMM.

**Results:**

This study aims to reconstruct the recent demographic histories of five cool-temperate tree species in Eastern Asia, *Quercus mongolica*, *Q. liaotungensis*, *Juglans cathayensis*, *J. mandshurica* and *J. ailantifolia*, by using 19 microsatellite markers with two methods considering generalized stepwise mutation model (GSM) (MIGRAINE and VarEff). Both programs revealed that all the five species experienced expansions after the Last Glacial Maximum (LGM). Within butternuts, *J. cathayensis* experienced a more serious bottleneck than the other species, and within oaks, *Q. mongolica* showed a moderate increase in population size and remained stable after the expansion. In addition, the point estimates of the multistep mutation proportion in the GSM model (*p*_GSM_) for all five species were between 0.50 and 0.65, indicating that when inferring population demographic history of the cool-temperate forest species using microsatellite markers, it is better to assume a GSM rather than a SMM.

**Conclusions:**

This study provides the first direct evidence that five cool-temperate tree species in East Asia have experienced expansions after the LGM with microsatellite data. Considering the mutation model of microsatellite has a vital influence on demographic inference, combining multiple programs such as MIGRAINE and VarEff can effectively reduce errors caused by inappropriate model selection and prior setting.

**Supplementary Information:**

The online version contains supplementary material available at 10.1186/s12862-021-01805-w.

## Background

Understanding the demographic history of species is a central issue in evolutionary ecology. At present, it is widely believed that repeated Quaternary glacial cycles since the Cenozoic, especially the Last Glacial Maximum (LGM, 20–18 kya [1]) [2–4], have strongly influenced the distribution and population dynamics of temperate tree species in the northern hemisphere [5–8]. In recent years, whole-genome sequencing coupled with new methods, such as the Sequentially Markovian Coalescent (SMC) based methods, e.g., the Pairwise Sequentially Markovian Coalescent (PSMC) model [9] and the Multiple Sequentially Markovian Coalescent (MSMC) model [10], and the site frequency spectrum (SFS) based method, e.g., stairway plot [11], contributes to illustrate the patterns of population demography. However, the SMC methods cannot efficiently reveal the continuous changes of population size within the last ten thousand years [12] and the SFS methods cannot provide an accurate estimation of population demography if the sample size is limited. However, sample size may be necessarily small for the projects of whole-genome sequencing in the non-model organisms [13]. Thus, to accurately predict recent population dynamics is still a major challenge for evolutionary ecologists, which requires molecular markers with much more polymorphisms. Microsatellites may be an option because of their high mutation rate [14, 15] of 10^–6^–10^–2^ per generation per locus [16, 17] and relatively selective neutrality [18, 19]. Moreover, considering there are thousands of studies that have employed microsatellite markers to study species evolution histories in the last two decades, it provides us opportunity to study population demography from these existing data.

Microsatellites have been widely used in population dynamics since the beginning of this century. Before the likelihood-based methods have been developed, most moment-based programs relied on summary statistics of genetic data and tested departure from theoretical distributions under a given demographic and mutational model, such as BOTTLENECK [20] and M-RATIO [21], which can only detect population declines. However, the moment-based methods suffer from a limited statistical power because they do not give any estimate of the severity and duration of the bottleneck. Likelihood-based methods such as MSVAR [22, 23] and MIGRATE [24] coupled with Monte Carlo sampling offer a powerful alternative to the moment-based methods [25–27], but they only take into account the strict stepwise mutation models (SMMs) [28], although many models have been developed to describe microsatellite mutation mechanisms [29]. It has been widely recognized that deviations from an SMM can cause false detection of population shrinkage [22, 30]. For these reasons, Leblois et al. [31] developed a program (MIGRAINE) which used a maximum-likelihood method to infer the past changes of population size with a generalized stepwise mutation model (GSM, [32]). Additionally, Nikolic and Chevalet [33] developed another program, VarEff, which used a composite-likelihood method to infer transient changes in population size in the past with multiple mutation models.

Ecologically dominant forest trees with wide distribution provide an excellent opportunity to explore the influences of climate and geography on their demographic histories [34]. In this study, we chose five temperate tree species distributed in East Asia, two oak species, *Quercus mongolica* and *Q. liaotungensis*, and three Asian butternut species, *Juglans cathayensis*, *J. mandshurica* and *J. ailantifolia*, to detect their recent population dynamics with microsatellite data. All five species are wind-pollinated, and have low to moderate genetic differentiation among populations [35–37]. For oaks, *Quercus mongolica* is mainly distributed in northeastern China [38], whereas *Q. liaotungensis* is divided into two clades, a northeast group scattered in the Changbai Mts. and partially distributed in North Korea and the Far East of Russia, and a northwest group mainly distributed in the Qinling, Liupan and Lüliang Mts. in northern China [39]. The historical population dynamics of *Q. mongolica* and *Q. liaotungensis* has not yet been reported. For butternuts, *J. mandshurica* is distributed in northeastern China, *J. cathayensis* occurs in southern China and Taiwan Island, and *J. ailantifolia* is only distributed in Japan [40]. Bai et al. [41] had estimated the divergence time and ancestral effective population size using DIYABC approach and microsatellite data, but the estimation of posterior parameters was greatly influenced by prior value setting [42, 43]. Later, Bai et al. [44] applied the PSMC method to the whole genome resequencing data to infer butternut population demography, but PSMC cannot reveal the population changes within the last ten thousand years because few coalescent events are expected to have occurred during this time period [9].

The main goal of this study was to reconstruct the recent demographic histories of the above-mentioned five temperate tree species with microsatellite data. To that end, we applied two methods implementing a GSM, i.e., MIGRAINE, which is based on the maximum likelihood method, and VarEff, which is based on approximate likelihood. Then, we assessed the effects of microsatellite mutation models and the multistep mutation proportions in the GSM on population demography inference and compared the advantages and disadvantages of the two programs. Our research can help to understand the recent demographic histories of temperate trees as well as shed some light on how microsatellite data can be used to analyze recent population size changes.

## Results

### Effective population size fluctuation

**Fig. 1:**
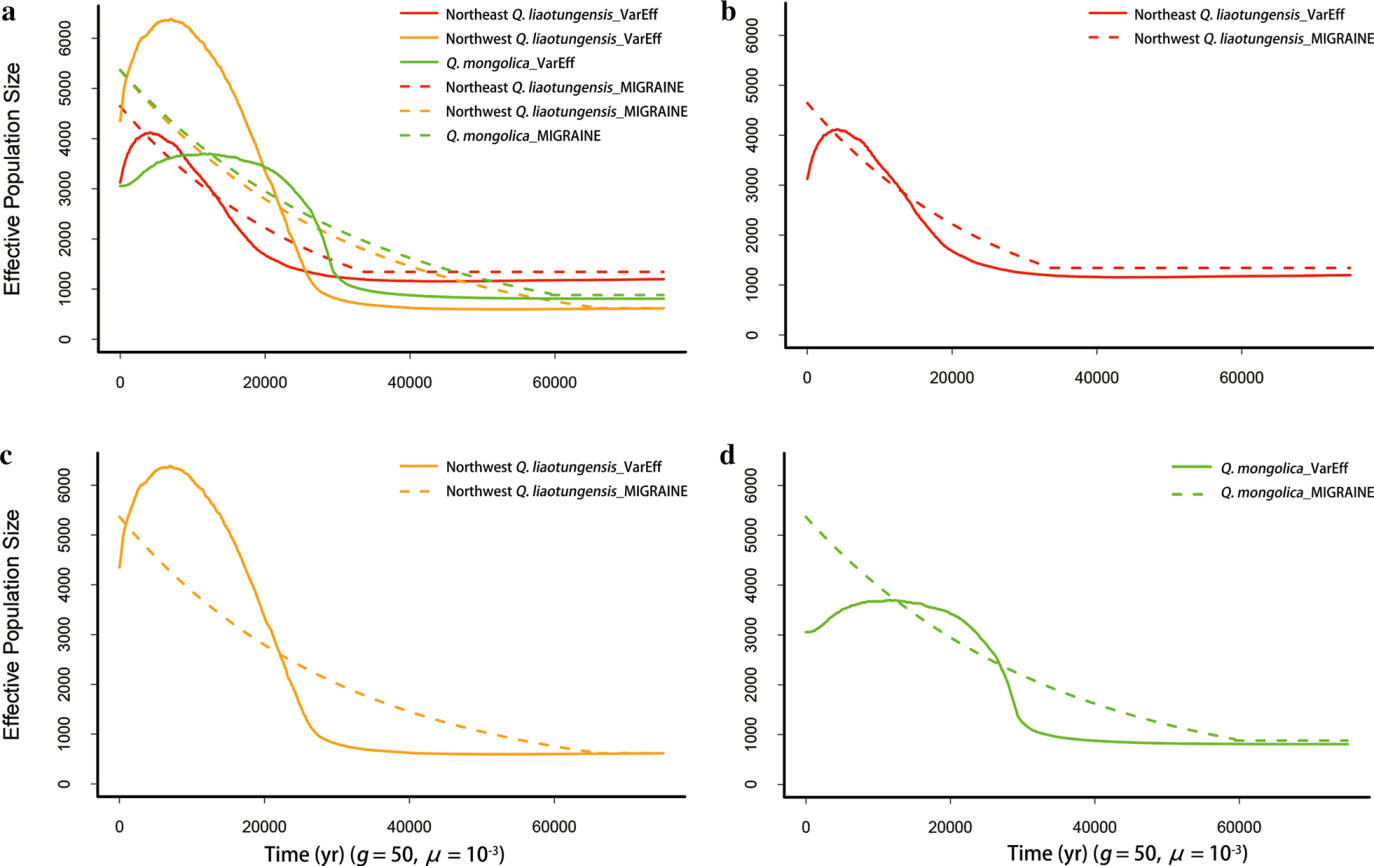
Population size fluctuation of two oak species. **a** All two species of oaks; **b** Northeast *Q. liaotungensis*; **c** Northwest *Q. liaotungensis*; **d** *Q. mongolica*. The solid line represents the results of VarEff, and the dotted line represents the results of MIGRAINE. *μ*, mutation rate per locus per generation. *g*, generation time (yr)

**Fig. 2:**
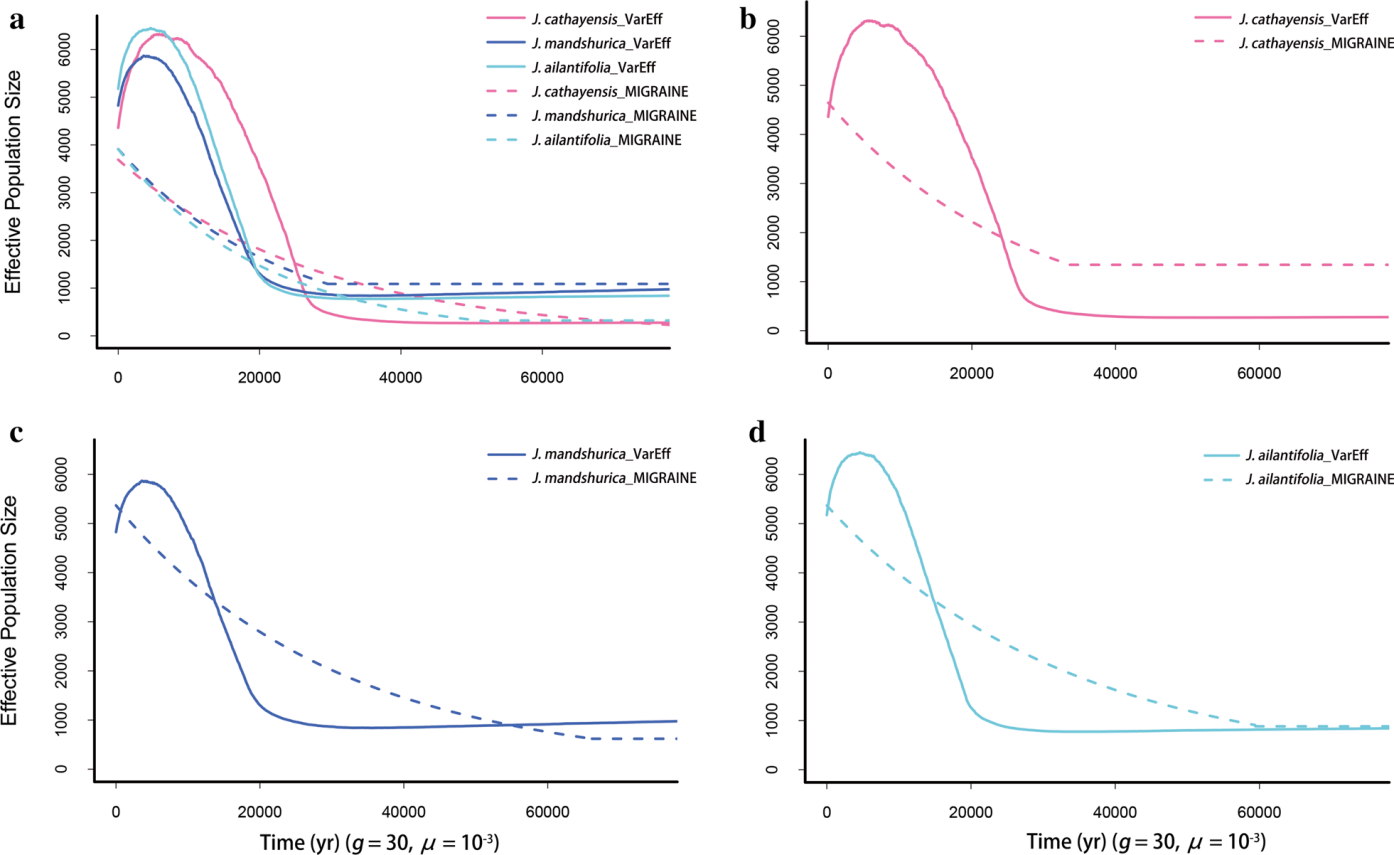
Population size fluctuation of three Asian butternut species. **a** All three species of Asian butternuts; **b** *J. cathayensis*; **c** *J. mandshurica*; **d** *J. ailantifolia*. The solid line represents the results of VarEff, and the dotted line represents the results of MIGRAINE. *μ*, mutation rate per locus per generation. *g*, generation time (yr)

Both MIGRAINE and VarEff were used to estimate the current and past effective population sizes and the *N*_e_ changing times in the two species of oaks and three species of Asian butternuts (see Table 1 and Additional file 1: Table S1). MIGRAINE and VarEff showed similar trends in the population dynamic curves for the five species, in which they experienced a rapid population expansion after LGM (Figs. 1, 2). However, the specific expansion times estimated from MIGRAINE and VarEff were different. Considering MIGRAINE can only deal with simple model and provide a wide confidence interval (Table 1), it would give a less precise value than VarEff. So, in the following, we focus our discussion on the VarEff results.

**Table 1:** Comparison of population parameters estimated using MIGRAINE and VarEff

Populations (sample size)	Methods	*p*_GSM_	*θ*	*θ*_anc_	*D* = *G* × *μ*	*N*_ratio_ = *θ*/*θ*_anc_	*N* (× 10^4^)	*N*_anc_ (× 10^4^)	*T*_years_ (kya)
Northeast *Q. liaotungensis* (139)	MIGRAINE	0.53 [0.45–0.60]	18.58 [13.55–27.93]	5.37 [2.07–9.11]	0.67 [0.25–1.80]	3.46 [1.74–8.16]	0.46 [0.34–0.70]	0.13 [0.05–0.23]	33.65 [12.55–89.75]
	VarEff		12.50	4.79	[0.20–0.40]	2.61	0.31	0.12	[10.00–20.00]
Northwest *Q. liaotungensis* (240)	MIGRAINE	0.58 [0.53–0.63]	21.47 [17.64–27.35]	2.48 [0.001–5.34]	1.32 [0.73–8.17]	8.67 [3.98–14,696.00]	0.54 [0.44–0.68]	0.06 [0.0003–0.13]	66.15 [36.30–40.845]
	VarEff		17.40	2.46	[0.30–0.40]	7.08	0.43	0.06	[15.00–20.00]
*Q. mongolica* (502)	MIGRAINE	0.62 [0.56–0.66]	20.88 [17.76–25.85]	3.52 [0.01–6.77]	1.19 [0.68–6.02]	5.93 [2.98–1314.00]	0.52 [0.44–0.65]	0.09 [0.0003–0.17]	59.45 [34.10–301.00]
	VarEff		12.23	3.24	[0.30–0.50]	3.78	0.31	0.08	[15.00–25.00]
*J. cathayensis* (596)	MIGRAINE	0.64 [0.60–0.68]	14.76 [12.41–17.60]	0.64 [0.02–4.08]	2.95 [1.06–9.72]	23.21 [3.58–569.60]	0.37 [0.31–0.44]	0.02 [0.0006–0.10]	88.41 [31.86–291.63]
	VarEff		17.44	1.11	[0.40–0.80]	15.72	0.44	0.03	[12.00–24.00]
*J. mandshurica* (399)	MIGRAINE	0.62 [0.56–0.66]	15.65 [12.69–20.46]	4.35 [0.82–8.50]	0.99 [0.29–3.36]	3.60 [1.81–17.41]	0.39 [0.32–0.51]	0.11 [0.02–0.21]	29.64 [8.61–168.20]
	VarEff		19.30	4.01	[0.30–0.60]	4.81	0.48	0.10	[9.00–18.00]
*J. ailantifolia* (107)	MIGRAINE	0.62 [0.56–0.66]	18.12 [14.02–24.39]	1.29 [0.13–5.01]	1.80 [0.68–5.08]	14.08 [3.67–141.50]	0.45 [0.35–0.61]	0.03 [0.003–0.13]	54.03 [20.28–152.25]
	VarEff		19.75	3.41	[0.30–0.60]	5.79	0.49	0.09	[9.00–18.00]

The estimates of past and current population sizes and times in years (*T*_years_) obtained by MIGRAINE and VarEff are converted with a fixed mutation rate of 10^–3^ mutation per locus per generation and a generation time of 50 years for oaks, 30 years for Asian butternuts. The population size calculated by VarEff is median. *θ*, scaled current effective population size by mutation rate; *θ*_anc_, scaled ancestor effective population size by mutation rate; *N*_ratio_ = *θ*/*θ*_anc_; *N*, current effective population size (individual number); *N*_anc_ ancestor effective population size (individual number); *G*, time measured by generations; *μ*, mutation rate per locus per generation

With a fixed mutation rate of 1 × 10^–3^ per locus per generation [16, 17] and a generation time of 50 years for oaks [45, 46] and 30 years for Asian butternuts [44], all five species began to expand at the time interval of 25–10 kya. Within oaks, the northwestern group of *Q. liaotungensis* had a high increase in population size (*N*_e_ ≈ 4,300), while the northeastern group of *Q. liaotungensis* and *Q. mongolica* showed a moderate increase (*N*_e_ ≈ 3,100). *Q. mongolica* remained stable size after the expansion at 25 kya (Fig. 1d). Within butternuts, *J. cathayensis* began to expand at 24 kya, while *J. mandshurica* and *J. ailantifolia* expanded at 18 kya (Fig. 2b). The effective population size of *J. cathayensis* (*N*_e_ ≈ 300) was much smaller than *J. mandshurica* and *J. ailantifolia* before expansion.

VarEff suggested all five species had experienced a population decline after their expansions. However, as pointed out by Nikolic et al. [33], it should be very cautious to make such a conclusion, because VarEff cannot distinguish well between ongoing expansion and finished expansion without *N*_*e*_ changes afterward. Both scenarios would be detected as a population expansion followed by a recent population decline.

### Effects of microsatellite mutation models on demographic inference

**Fig. 3:**
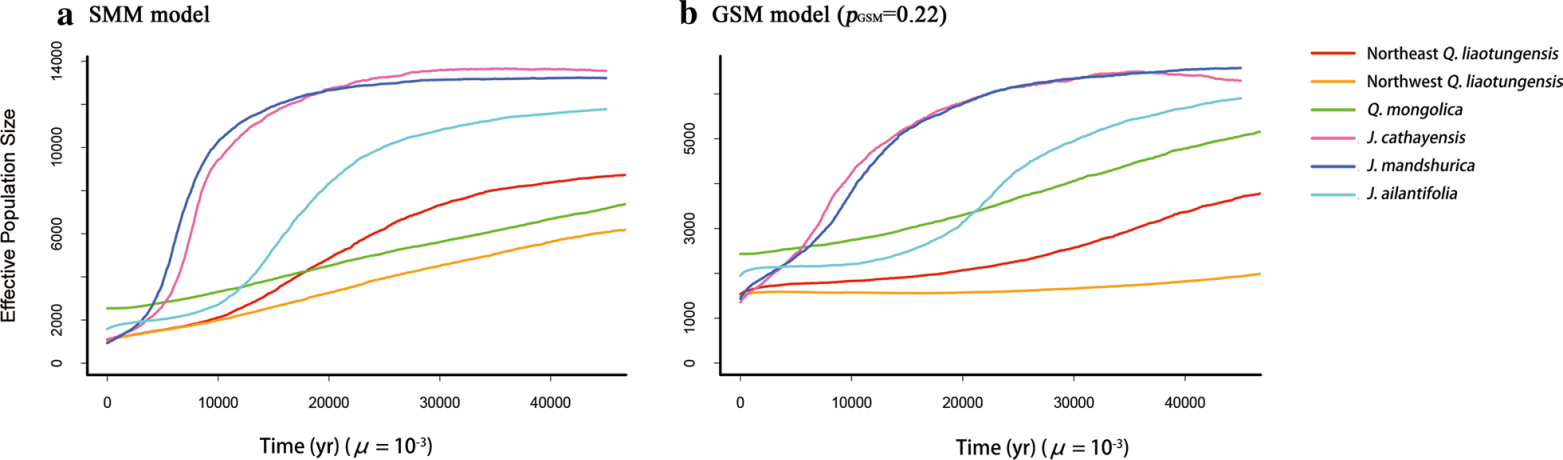
Effects of microsatellite mutation models on demography inference. **a** SMM model; **b** GSM model with the proportion of multi-step mutation as 0.22. *μ*, mutation rate per locus per generation. The generation times were set as 50 years for oaks [45, 46] and 30 years for Asian butternuts [44]

We used MIGRAINE to estimate the multistep mutation proportions (*p*_GSM_) in the GSM for the five species. The point estimates for *p*_GSM_ were quite high, at the range of 0.53–0.63 for oaks and 0.62–0.64 for butternuts (Table 1). Then, we implemented VarEff to assess the effects of *p*_GSM_ on the demographic inference of these trees. When *p*_GSM_ was set as 0.22, all the five species were detected to experience population declines (Fig. 3b). Moreover, if we set mutation model as SMM instead of GSM, we also got a result of population decline (Fig. 3a). These findings indicate that we will get a wrong signal of population dynamics if we misuse the mutation model or incorrectly set the *p*_GSM_ parameter.

## Discussion

### Historical population dynamics is driven by climate fluctuations

The repeated Quaternary glacial cycles had a profound impact on the geographic history of temperate plants, especially during LGM (20–18 kya) [2–4]. During glacial periods, temperate trees were either forced to retreat southward [47] or restricted to a few northern refugia [48–52] with dramatic population declines. During interglacial periods, they recolonized their modern distribution areas with population expansion. After analyzing the microsatellite data of the five tree species with two complementary programs, we found that they all experienced a strong and recent population expansion since the LGM (Figs. 1,2, Table 1). This finding accords well with what ecological niche modeling predicted for Asian butternuts [41] and oaks [53], that is, the present distribution of butternuts and oaks are much wider and larger than that in LGM (see Fig. 6 of [41] and Fig. 4 of [53]). Since the expansion times of the five species were highly coincident with each other, we believe that their population dynamical changes are most likely attributed to climate fluctuations since the LGM.

Among the five species, *J. cathayensis* has experienced the most serious bottleneck, leading to a smallest effective population size before expansion. This indicated that *J. cathayensis* may be more susceptible to environmental changes than the other species. According to VarEff, *J. mandshurica* and *J. ailantifolia* had similar population sizes before and after their expansions, but different from that of *J. cathayensis*, which is consistent with the results of PSMC [44] that *J. cathayensis* decreased faster to an extremely small size than *J. mandshurica* and *J. ailantifolia* before LGM. The plot of *J. cathayensis* is different from those of *J. mandshurica* and *J. ailantifolia* before and after LGM, and *J. mandshurica* and *J. ailantifolia* were highly coincident with each other for most of time (Fig. 2). Among the three species of oak, population dynamics of the two groups of *Q. liaotungensis* were very similar, but different from *Q. mongolica* which maintained a constant size after the expansion. This may be because *Q. mongolica* is more acclimated to cold but not to the dry warming climate [54].

### Effects of the microsatellite mutation model on inferring population dynamics

The mutation model of microsatellite is important for the estimation of population parameters such as the number of migrants, divergence time and *N*_e_ [17]. Although an increasing amount of data has shown that most microsatellite loci conform to a GSM rather than an SMM [55, 56], many programs for analyzing microsatellite data still assumed SMM as the only model. For example, as one of the most commonly used software in the last two decades, MSVAR includes only SMM model [22]. However, the misuse of SMM might generate severe bias in the inference of demographic history [30]. This is because multistep mutations in the GSM model can produce a pattern of allele length distribution very similar to that of population decline under the SMM model [31, 57]. Moreover, when inferring the ancient evolution events, the error rate of population parameter estimation with microsatellites is much higher than that with SNPs if an SMM or an infinite allele model (IAM) is assumed [14]. Therefore, when we are not sure about the microsatellite mutation process in a species, we should be very cautions to choose SMM as the mutation model [15]. In our study, the proportions of multistep mutations in the GSM calculated by MIGRAINE ranged from 0.50 to 0.65 in the oaks and 0.60 to 0.65 in the Asian butternuts, indicating GSM model was more suitable for microsatellites, at least for cool-temperate tree species. Bai et al. [41] estimated a similar value for Asian butternuts by using DIYABC program. We also found that different settings for the proportions of multistep mutations may lead to completely opposite trends of population size change, suggesting that correctly setting this parameter of the GSM (Fig. 3) is very important.

There are thousands of studies that have employed microsatellite markers in the last two decades, and hence it is worth continuing to mine the information from these existing data. As we noted earlier, microsatellites are well suited to detect recent population changes due to their high mutation rate and neutrality [14, 15]. Therefore, they can provide a good complement to the programs analyzing whole genome data which often cannot estimate recent population dynamics [9]. The development of bioinformatic methods in recent years will allow us to understand more about the distribution and mutation mechanisms of microsatellites [17]. For all of these reasons, microsatellites would remain an effective and cost-efficient marker to study population demography in the genomic age [58].

### Comparison of MIGRAINE vs. VarEff

MIGRAINE is a software that relies on the importance sampling of gene genealogies and the coalescent theory under the maximum likelihood framework to estimate current and past *N*_e_, as well as the time of changes in population size. It is more flexible and robust than other programs because it can accommodate several demographic models, e.g., isolation by distance model (*IBD*), the single population dynamic variation model (*OnePopVarSize*), the founder model (*OnePopFounderFlush*) and several microsatellite mutation models, e.g., SMMs, GSMs, and infinite sites mutation models (ISMs). In addition, MIGRAINE can estimate the multistep mutation proportion (*p*_GSM_) in the GSM, which is a key parameter for other programs (e.g., VarEff). However, MIGRAINE is time consuming, and when running models of *OnePopVarSize* or *OnePopFounderFlush*, more runs (usually > 2000) per sampling point are needed. Furthermore, MIGRAINE assumes that a single isolated population has undergone a single past size change, which definitely oversimplifies actual complex population dynamics.

Compared to MIGRAINE, VarEff relies on an approximation likelihood algorithm to detect population demography. So, it need not presuppose any scenario about demographic history, such as monotonous growth or decline. VarEff is less dependent on the priors and can evaluate the effects of different mutation models on the results [33]. VarEff has included several microsatellite mutation models, such as SMMs, two-phase models (TPMs) and GSMs, but needs to set the multistep mutation proportion parameter in the GSM. For VarEff, the main disadvantages are that it cannot infer ancient population changes (e.g., *G* × *μ* > 20) and would give a false population decline when a SMM model was wrongly chosen or when substantial gene flow exists.

In view of their advantages and disadvantages, we advocate that researchers should apply MIGRAINE and VarEff in combination when inferring population demography with microsatellite data, which can effectively reduce errors caused by inappropriate model selection and prior parameter settings.

## Conclusions

Even though whole-genome sequencing is prevalent today, the low mutation rate of SNPs and the limited sampling size are two main hurdles preventing us from inferring recent population dynamics accurately. In this context, microsatellites may be a good choice for reconstructing population dynamics due to their high mutation rates and selective neutrality. Moreover, researchers have accumulated much more microsatellite data in last two decades, which can be reused to help infer most recent demographic events. Indeed, using microsatellites data we have successfully revealed expansions among five cool-temperate tree species in East Asia after the Last Glacial Maximum. This confirms previous hypothesis that the distribution range of temperate trees have expanded with the climate warming during interglacial period [41, 53]. Additionally, we have shown that when inferring demographic history with microsatellite data, both the microsatellite mutation model and the parameter settings can have a significant influence on the result. As a rule of thumb, it is better to assume a GSM rather than a SMM and to apply multiple programs to carry out microsatellite analysis.

## Methods

### Data source

All data were from previous studies; the data on oaks came from Zeng et al. [39] and the data on Asian butternuts from Bai et al. [41]. In both studies, 19 microsatellite markers were used respectively to generate polymorphism data.

**Fig. 4:**
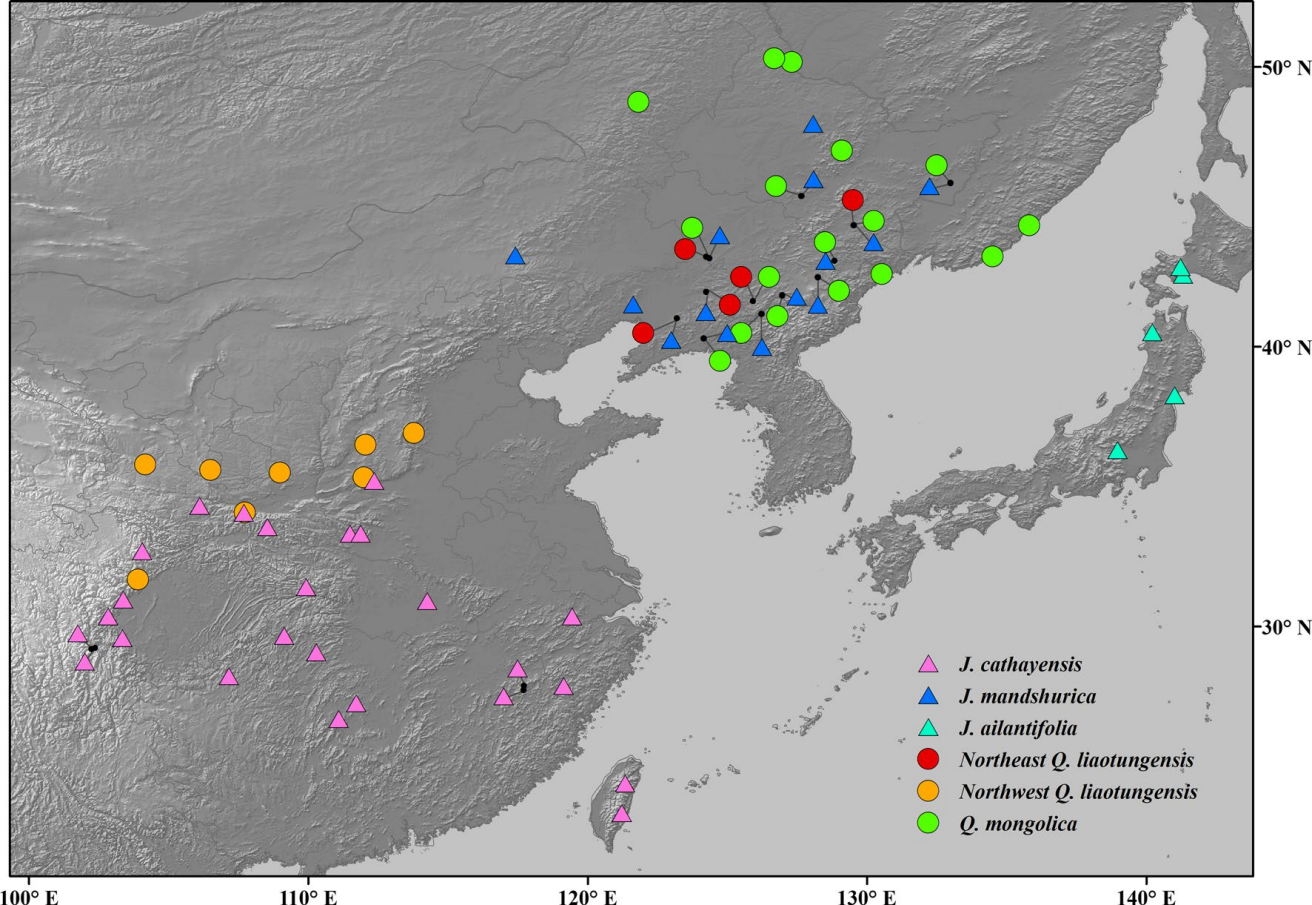
Geographic distribution of two species of oaks and three species of Asian butternuts, according to Zeng et al. [39] and Bai et al. [41]

Based on previous studies, the investigated areas covering the whole range of *Q. mongolica* and most distribution areas of *Q. liaotungensis* in China*,* included 502 individuals from 17 *Q. mongolica* populations, 139 individuals from five populations of northeast *Q. liaotungensis* and 240 individuals from eight populations of northwest *Q. liaotungensis*. The sample distribution range of Asian butternuts also covered the entire distribution area of the three species, including 596 individuals from 25 *J. cathayensis* populations, 399 individuals from 14 *J. mandshurica* populations and 107 individuals from five *J. ailantifolia* populations (Fig. 4).

### Data analysis

#### MIGRAINE analysis of historical population dynamics

We explored the demographic history of the above five species using MIGRAINE 0.5.4 (http://kimura.univ-montp2.fr/~rousset/Migraine.htm), which is based on importance sampling of gene genealogies under a maximum likelihood framework. It is extended for GSM which notably allows departure from the strict SMM with the parameter *p*_GSM_ for the geometric distribution of mutation sizes. The present and ancestral scaled population sizes in mutation rate (*θ* = 4*Nμ*, *θ*_anc_ = 4*N*_anc_*μ*), and the occurrence time of the past change scaled by mutation rate (*D* = *G* × *μ*) were calculated. Here, *N*_anc_ is the ancestral effective population size and *N* is the current effective population size; *μ* is the mutation rate per locus per generation; *G* is the time measured by generations. The detection of significant past change in the population is based on the population size ratio (*θ*_ratio_ = *θ*/*θ*_anc_). If *θ*_ratio_ < 1, the population has experienced contraction; if *θ*_ratio_ > 1, the population has experienced expansion. Also, we estimated the multistep mutation proportion of the GSM (*p*_GSM_) using MIGRAINE.

The *OnePopVarSize* model was used, which considers a single isolated population with a unique past size change. To ensure enough points with high likelihoods for the smoothing procedure, we considered relatively high values of parameter *NRunsPerPoint*. For the data of northeast *Q. liaotungensis*, northwest *Q. liaotungensis*, *Q. mongolica* and *J. mandshurica*, we considered 2,000 trees, with 200 points in each iteration and a total of 16 iterations. For the data of *J. cathayensis* and *J. ailantifolia*, MIGRAINE was run using 20,000 trees, with 200 points in each iteration and a total of 16 iterations.

#### VarEff analysis of historical population dynamics

Since MIGRAINE infers population dynamics under a model of a single panmictic population with one exponential change in population size, the highly simplified demographic model may be insufficient to characterize the actual situation. So, we also used VarEff [33], a program estimating the past changes in *N*_e_ by using approximate likelihoods under a MCMC approach. It has been found to be especially useful for providing evidence of transient changes in population size in the past. The VarEff method was implemented in the *R* package VarEff (https://qgsp.jouy.inra.fr). By using the functions in the VarEff package, we extracted several global statistics of *N*_e_ (arithmetic and harmonic means, mode, median, and quantiles). Since the median of the posterior distribution was found to be the most robust estimator [33], we visualized it at different times in the past.

Using the GSM as the microsatellite mutation model, we first set the multistep mutation proportion, *C* (which is the same as parameter *p*_GSM_ in MIGRAINE) as that estimated by MIGRAINE. In order to compare with the results of MIGRAINE, we set the parameter *TMAX* (Length of the period for which the distributions of *N*_e_ in the past) in VarEff with reference to the estimates of parameter *D* in MIGRAINE. For the parameter settings related to MCMC, *NumberBatch*, *LengthBatch*, and *SpaceBatch* were set to be 10,000, 1, and 100, respectively, and *burnin* was set to 10,000.

## Supplementary Information

**Additional file 1: Table S1.** Detailed population parameters estimated by VarEff. *θ (*= 4*Nμ)*, the scaled current effective population size in mutation rate; *θ*_anc_ *(*= 4*N*_anc_*μ)*, the scaled ancestor effective population size in mutation rate; *θ*/*θ*_anc_ (*H*), the ratio of harmonic means of the effective population size; *θ*/*θ*_anc_ (*M*), the ratio of medians of the effective population size. *N*, the current effective population size (individual number); *N*_anc_, the ancestral effective population size (individual number); *μ*, the mutation rate per locus per generation.

## Data Availability

The datasets analyzed during the current study (SSR genotypes and geographical locations of collections) are available in the Dryad Digital Repository, 10.5061/dryad.83bk3j9mz.
