# Supplementary material for: Recent demographic histories of temperate deciduous trees inferred from microsatellite markers
Source: BMC Ecol Evol. 2021 May 18;21:88. doi: 10.1186/s12862-021-01805-w (PMC8130339; doi:10.1186/s12862-021-01805-w)
Supplement: Supplementary file 1 — Additional file 1: Table S1. Detailed population parameters estimated by VarEff. θ (= 4Nμ), the scaled current effective population size in mutation rate; θanc (= 4Nancμ), the scaled ancestor effective population size in mutation rate; θ/θanc (H), the ratio of harmonic means of the effective population size; θ/θanc (M), the ratio of medians of the effective population size. N, the current effective population size (individual number); Nanc, the ancestral effective population size (individual number); μ, the mutation rate per locus per generation. [file 12862_2021_1805_MOESM1_ESM.docx]

**Additional file 1**

**Article title** Recent demographic histories of temperate deciduous trees inferred from microsatellite markers

Authors Yu Cao^1^, Da-Yong Zhang^1^, Yan-Fei Zeng^2^ and Wei-Ning Bai^1*^

Table S1. Detailed population parameters estimated by VarEff. *θ (=*4*Nμ)*, scaled current effective population size by mutation rate; *θ*_anc_ *(=*4*N_anc_μ)*, scaled ancestral effective population size by mutation rate; *θ*/*θ*_anc_ (H), the ratio of harmonic means of effective population size; *θ*/*θ*_anc_ (M), the ratio of median of effective population size. *N*, current effective population size (individual number); *N*_anc_ ancestral effective population size (individual number); *μ*, mutation rate per locus per generation.

| Populations | Oaks | | | | | | |  | Asian butternuts | | | | | |
| --- | --- | --- | --- | --- | --- | --- | --- | --- | --- | --- | --- | --- | --- | --- |
|  | Northeast *Quercus liaotungensis* | | Northwest *Q. liaotungensis* | | | *Q. mongolica* | |  | *Juglans cathayensis* | | *J. mandshurica* | | *J. ailantifolia* | |
| Effective population size | *θ* | *θ*_anc_ | *θ* | *θ*_anc_ | *θ* | | *θ*_anc_ |  | *θ* | *θ*_anc_ | *θ* | *θ*_anc_ | *θ* | *θ*_anc_ |
| Arithmetic means | 28.74 | 10.48 | 37.64 | 6.10 | 14.47 | | 3.33 |  | 34.24 | 7.18 | 36.90 | 14.37 | 39.25 | 10.36 |
| Harmonic means | 5.76 | 4.48 | 6.83 | 2.00 | 10.40 | | 3.08 |  | 6.91 | 0.67 | 9.17 | 2.91 | 7.22 | 2.78 |
| Mode | 6.53 | 4.57 | 6.99 | 2.20 | 9.09 | | 3.06 |  | 9.78 | 0.90 | 11.45 | 2.99 | 9.35 | 2.96 |
| Median | 12.50 | 4.79 | 17.40 | 2.46 | 12.23 | | 3.24 |  | 17.43 | 1.11 | 19.30 | 4.01 | 19.75 | 3.41 |
| *θ*/*θ*_anc_ (H) | 1.28 | | 3.42 | | | 3.38 | |  | 10.35 | | 3.15 | | 2.92 | |
| *θ*/*θ*_anc_ (M) | 2.61 | | 7.08 | | | 3.78 | |  | 15.72 | | 4.81 | | 5.70 | |
